# Are ketogenic diets promising for Alzheimer’s disease? A translational review

**DOI:** 10.1186/s13195-020-00615-4

**Published:** 2020-04-14

**Authors:** Matthieu Lilamand, Baptiste Porte, Emmanuel Cognat, Jacques Hugon, François Mouton-Liger, Claire Paquet

**Affiliations:** 1 INSERM U1144 Optimisation Thérapeutique en Neuropsychopharmacologie, Université de Paris, Paris, France; 2 Centre de Neurologie Cognitive/CMRR Paris Nord Ile de France, APHP Nord Université de Paris, Lariboisière Hospital 200, rue du Faubourg Saint Denis, 75010 Paris, France; 3 Department of Geriatrics, Bichat and Bretonneau Hospitals, APHP Nord Université de Paris, 75018 Paris, France

**Keywords:** Ketogenic diet, Alzheimer’s disease, Brain metabolism, Animal models

## Abstract

**Background:**

Brain amyloid deposition and neurofibrillary tangles in Alzheimer’s disease (AD) are associated with complex neuroinflammatory reactions such as microglial activation and cytokine production. Glucose metabolism is closely related to neuroinflammation. Ketogenic diets (KDs) include a high amount of fat, low carbohydrate and medium-chain triglyceride (MCT) intake. KDs lead to the production of ketone bodies to fuel the brain, in the absence of glucose. These nutritional interventions are validated treatments of pharmacoresistant epilepsy, consequently leading to a better intellectual development in epileptic children. In neurodegenerative diseases and cognitive decline, potential benefits of KD were previously pointed out, but the published evidence remains scarce. The main objective of this review was to critically examine the evidence regarding KD or MCT intake effects both in AD and ageing animal models and in humans.

**Main body:**

We conducted a review based on a systematic search of interventional trials published from January 2000 to March 2019 found on MEDLINE and Cochrane databases. Overall, 11 animal and 11 human studies were included in the present review. In preclinical studies, this review revealed an improvement of cognition and motor function in AD mouse model and ageing animals. However, the KD and ketone supplementation were also associated with significant weight loss. In human studies, most of the published articles showed a significant improvement of cognitive outcomes (global cognition, memory and executive functions) with ketone supplementation or KD, regardless of the severity of cognitive impairments previously detected. Both interventions seemed acceptable and efficient to achieve ketosis.

**Conclusion:**

The KD or MCT intake might be promising ways to alter cognitive symptoms in AD, especially at the prodromal stage of the disease. The need for efficient disease-modifying strategies suggests to pursue further KD interventional studies to assess the efficacy, the adherence to this diet and the potential adverse effects of these nutritional approaches.

## Background

Carbohydrates represent the primary energy source for the brain. However, when glucose is not readily available (e.g. starvation), a metabolic switch occurs in favour of ketone bodies (KB), usually released by the liver. The diets specifically designed for KB production are called ketogenic diets (KDs) [1, 2]. They are the first and only nutritional interventions that enabled a significant reduction in the incidence of seizures in pharmacoresistant epilepsy [3] or in chronic cluster headaches [4]. The core characteristics of the KD are the association of a high amount of fat, with low carbohydrate intake, usually a macronutrient ratio of fat to protein and carbohydrate combined equal to 3–4:1. Alternative KDs were developed using ketone supplementation (KS), in which fats are provided with medium-chain triglyceride (MCT) intake. Their common objective is to achieve ketosis, leading to reduced insulin secretion and glycaemia within 48 h, as well as a shift in the brain’s metabolism [2].

In Alzheimer’s disease (AD), it is described numerous interrelations between abnormal glucose metabolism and the occurrence of brain lesions. First, AD could be a partial consequence of insulin resistance, which affects insulin signalling and favours in the brain abnormal deposition of β-amyloid peptide (Aβ) and phosphorylated tau (pTau) accumulation, leading in turn to cognitive decline [5]. Furthermore, the expression of apolipoprotein E allele 4 (*APOE4*) is a common risk factor for AD and for type 2 diabetes suggesting a common pathophysiological background [6]. As demonstrated by fluorodeoxyglucose positron emission tomography (FDG-PET), abnormal brain glucose metabolism in the temporal and parietal lobes occurs from the earliest stages in AD animal models and AD patients but also in asymptomatic individuals at risk for AD [7]. Interestingly, these hypometabolic regions are still able to take up KB, even though they can no longer utilize glucose [8].

At the cellular level, several KD neuroprotective effects have been observed, linked to various mechanisms: (i) reduction of the concentration of several excitatory neurotransmitters (e.g. glutamate) [9], (ii) stabilization of synaptic functions due to enhanced mitochondrial biogenesis [10] and (iii) reduction of reactive oxygen species generation and increase adenosine triphosphate availability [11]. Moreover, KB have specific protective effects against cerebral Aβ toxicity and cell damage as shown in rat cultured hippocampal neurons [12]. Aβ can induce neuroinflammation, which is a current therapeutic target in AD. KD could modulate Aβ toxicity by promoting the action of endogenous anti-inflammatory molecules such as peroxisome proliferator-activated receptor γ, leading to a decreased systemic inflammation [13].

These observations, as well as the current lack of success of anti-AD therapies or nutritional interventions to change the course of AD, suggest that KD or KS might be of therapeutic interest in these patients. A potential benefit of KDs in AD was already claimed in media and some scientific reports [14, 15]. However, as few works have brought about a critical review of existing data, we hereby propose a comprehensive and translational review of KD efficiency in both preclinical and clinical AD or ageing studies. Thus, the main objective of this review was to assess the effects of ketogenic interventions on clinical and metabolic outcomes (e.g. cognitive function, brain metabolism) or AD biomarkers, both in experimental animals and in humans. We will also examine the potential side effects of ketogenic interventions in these populations, in terms of nutritional change and adverse effects.

## Research process

We performed a systematic search in accordance with the Preferred Reporting Items for Systematic Reviews and Meta-Analyses (PRISMA) guidelines [16]. We identified all published articles between January 2000 and March 2019 on MEDLINE and Cochrane databases using the Medical Subject Heading (MeSH) terms “ketogenic diet” or “medium-chain triglyceride” and assorted combinations of the following terms: “Alzheimer’s disease”, “Alzheimer”, “cognition” and/or “memory”. We only included interventional studies using either KD or KS. We have excluded studies published in languages other than English, focusing on KD diet effects in diseases other than AD or performed in cellular models. Titles and abstracts were the base of the initial screening. We evaluated the eligibility of selected articles after full-text readings. We also examined all papers cited in the selected articles. We added additional references, based on their originality and/or relevance regarding the scope of this review. Data extraction was performed by six authors (ML, BP, EC, FML, JH, CP), using a standardized extraction form. This tool assessed the study design, population (number of animals/participants, animal model/ageing individuals or AD patients), type of ketogenic intervention, outcomes (e.g. biological endpoint, clinical endpoint, neuroimaging endpoint), follow-up duration, nutritional changes and potential side effects due to the intervention. This search was carried out in April 2019.

## Results

**Fig. 1:**
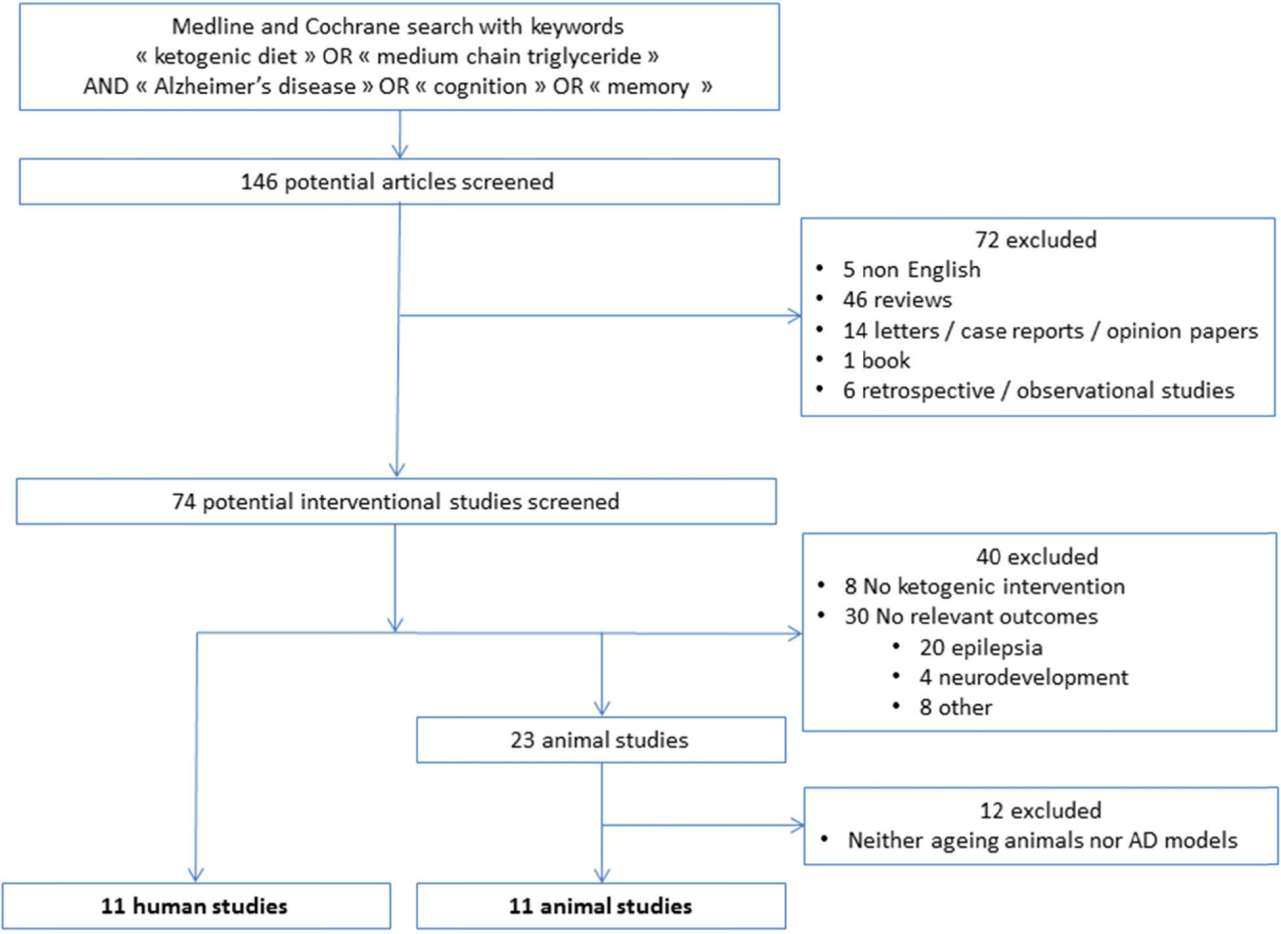
Flow diagram of studies selection

Among one hundred forty-six selected papers, we identified 84 interventional studies. Two additional interventional studies were found through the bibliography of the relevant animal studies papers. In total, 22 (11 animal studies and 11 human studies) were considered relevant for the present review. We summarized details of animal and human studies in Tables 1 and 2, respectively. Figure 1 outlines the results of the systematic searches.

**Table 1:** Animal studies discussed in the present review

Study 1st author (ref)	Models	Intervention	Number	FU	Outcomes	Positive results (intervention group)	Nutritional changes (vs ctrl group)
Aso [17]	APP/PS1 mice	KD + triheptanoin vs KD	28	12	Cognition, AD features inflammation markers	Cognition and neuroinflammation	Unassessed
Beckett [18]	APP/PS1 mice	KD ad libitum	65	4	Motor functions, AD features, oxidative stress	Motor coordination (rotarod)	Weight loss
Brownlow [19]	APP/PS1 and Tg4510 mice	KD ad libitum	60	16	Cognition, motor unctions, AD features	Motor coordination (rotarod)	Stable weight
Hernandez [20]	Young vs old WT rats	KD cal ctrl	56	12	Body composition, transporter expression	Less adipose tissues and reversed age-related transporters evolution	Unassessed
Hernandez [21]	Young vs old WT rats	KD cal ctrl	39	12	Cognition, transporter expression	Cognition and reversed age-related transporters evolution	Weight loss in old rats
Kashiwaya [22]	3xTgAD mice	KS vs CD	30	32	Cognition, AD features	Cognition and AD features	Weight loss
Newman [23]	Young vs old WT mice	Cyclic KD ad libitum	58	72	Cognition, motor functions, health span, gene expression	Cognition and reduced mid-life mortality	Stable weight
Pan [24]	Old beagle dogs	KS vs CD	24	32	Cognition	Cognition	Stable weight
Pawlosky [25]	3xTgAD mice	KS vs CD	24	32	Metabolism, protein expression	Reduced protein oxidation and BACE 1 expression	Weight loss
Van der Auwera [26]	APP/V717I mice	KD ad libitum, crossover	16	6	Cognition, AD features	Reduced brain total Aβ	Weight loss
Wang [27]	Old WT rats	KS (MCT 8 or 10) vs CD	36	8	Cognition, synapse pathway	Cognition and synaptic stability	Weight loss

*Aβ* beta-amyloid peptide, *AD* Alzheimer’s disease, *BACE 1* beta-secretase 1, *cal ctrl* calorie-controlled, *CD* control diet, *ctrl* control, *FU* follow-up duration (given in weeks), *KD* ketogenic diet, *KS* ketone supplementation

**Table 2:** Human studies discussed in the present review

Study 1st author (ref)	Population	Mean age (SD)	Intervention	*N*	Design	FU	Outcomes	Positive outcomes (intervention group)	Plasma KB levels	Nutritional changes	Side effects
Fortier [28]	MCI	75.4 (6.6)	KS vs placebo	52	RCT	26	MMSE, MoCa, brain PET imaging	Imaging (but not cognition)	Yes	Stable weight	No adverse effect
Ota [29]	Mild-moderate AD	73.4 (6.0)	KS vs control	20	RCT	12	Memory, executive function	Cognition	Yes	Unassessed	Unassessed
Torosyan [30]	Mild-moderate AD	79.9 (9.2)	KS vs control	14	RCT	6	Brain PET imaging	Imaging	No	Unassessed	Unassessed
Taylor [31]	MCI or AD dementia	73.1 (9.0)	KS	15	No control group	12	ADAS-cog, MMSE, feasibility	Cognition	Yes	Stable weight, cholesterol, glucose	Unassessed
Abe [32]	Nursing home residents	86.6 (4.8)	KS + vitD + leucine vs vitD leucine vs control	38	RCT	12	MMSE	Cognition	No	Improved muscle mass and function	Unassessed
Ota [33]	60+ adults no dementia	66.1 (2.9)	KS vs placebo	19	Crossover, RC	0	Memory, executive function	Cognition	Yes	Unassessed	Unassessed
Ohnuma [34]	Mild moderate AD	63.9 (8.5)	KS vs control	22	RCT	12	MMSE	None	Yes	Unassessed	Unassessed
Rebello [35]	MCI	Not available	KS vs control	6	RCT	24	ADAS-cog, TMT, DST	Inconclusive	Yes	Stable weight	No adverse effect
Krikorian [36]	MCI	70.1 (6.2)	KD vs CD	23	RCT	6	Executive function, memory	Cognition	No (urinary tests)	Weight loss	No adverse effect
Henderson [37]	Mild-moderate AD	76.9 (7.9)	KS vs placebo	152	RCT	12	ADAS-cog, ADCS-CGIC	Cognition	Yes	Unassessed	Frequent GI symptoms
Reger [38]	MCI or AD dementia	74.7 (6.7)	KS vs placebo	20	Crossover, RC	0	ADAS-cog, MMSE, word recall, Stroop	Cognition	Yes	Unassessed	Unassessed

*AD* Alzheimer’s disease, *ADAS-cog* Alzheimer Disease Assessment Scale, *ADCS-CGIC* Alzheimer’s Disease Cooperative Study Clinical Global Impression of Change, *CD* control diet, *DST* Digit Symbol Test, *FU* follow-up duration, *GI* gastrointestinal, *KB* ketone bodies, *KD* ketogenic diet, *KS* ketone supplementation, *MCI* mild cognitive impairment, *MoCA* Montreal Cognitive Assessment, *N* number, *MMSE* Mini-Mental Status Examination, *PET* positron emission tomography, *RCT* randomized controlled trial, *TMT* Trail Making Test, *vitD* vitamin D

## Preclinical studies

Two types of interventions were used: KD (*N* = 7) either ad libitum or calorie-controlled, and KS (*N* = 4), compared to control diet or placebo, respectively. Even if the total number of animals varied from one study to the other (from 16 to 65 with a mean of 48.5 ± 5.2 animals), the number in each treatment group was nearly the same. Overall, baseline age at the start, species or AD model type, sex and follow-up duration varied between studies.

### Ageing animals

Five studies assessed ketogenic interventions in ageing animals. The mean age at baseline varied depending on the studied species: 9 years for beagle dogs (*N* = 1), above 20 months for wild-type (WT) rats (*N* = 3) and 12 months for WT mice (*N* = 1). Only two studies out of five compared young (4-month-old WT rats) vs ageing animals [20, 21]. Three studies used only ageing male rodents [20, 23, 27] whereas two other studies compared the effect of KD/KS on ageing males and females [21, 24]. The follow-up duration was different according to species: 8 or 12 weeks for WT rats [20, 21, 27], 32 weeks for beagle dogs [24] and 72 weeks for old WT mice on which the effect of KD on life span was evaluated [23].

### Alzheimer’s disease animal models

Six studies included different AD mouse models, based on the amyloid cascade hypothesis. Briefly, 3xTgAD mice [22, 25], APP/PS1 mice [17, 18], APP [V7171] mice [26] and APP+PS1 [19] mice were used. Interestingly, Brownlow et al. choose to compare an amyloid-based AD model (APP+PS1 mice) and a tau-based AD model (Tg4510 AD mice) [19]. One of these studies used only female AD mouse model (APP [V7171]) [26]. Regarding baseline age, 3xTgAD mice were older (8.5 months) than APP [V7171] (3 months), APP/PS1 (between 1 and 3 months) and APP+PS1 and Tg4510 mice (5 months). The follow-up duration varied from 4 to 32 weeks (mean 17 ± 5.05 weeks) without a link between duration and a type of AD model.

### Outcomes and results

The following objectives were assessed: efficacy on cognition and/or motor functions (*N* = 3 assessing both, *N* = 5 assessing cognition only, *N* = 1 assessing motor function only), impact on neuropathological AD lesions (*N* = 5), KD safety (*N* = 1) and weight variations (*N* = 9). Eight studies (one in old WT mice, two in young vs ageing WT rats and five in AD mouse models) evaluated multiple outcomes.

### In ageing animals

Three studies evaluated the effects of KD in ageing animals, either on safety and cognition [23], weight and neurotransmitter function [20] or cognition/motor function and neurotransmitter function [21].

#### Effects of KD/KS on life expectancy and mortality

Only Newman et al. conducted a long-term study of KD. They showed that KD was able to reduce mid-life mortality but without improving maximum lifespan in old C57Bl6 WT mice [23].

#### Cognitive and motor impact of KD/KS

Four studies evaluated the cognitive impact of KD and showed a positive effect on cognition, in ageing animals [20, 23, 24, 27]. This effect was observed with several KD formulae (two calorie-controlled KD, two KS). This result suggests that brain metabolism can shift towards the use of KB, regardless of the kind of ketone-oriented diet used, with potential cognitive gain. Similarly, motor performances were improved in all studies, regardless of potential associated cognitive benefits. These results are consistent with many mouse studies demonstrating that keto-adaptation enhanced the capacity to transport and metabolize fat as well as motor abilities and recovery [39–41].

#### Mechanisms underlying cognitive effects of KD and KS

Hernandez et al. studied the effect of KD on transport protein expression in the brain of ageing mice, notably vesicular glutamate and gamma-aminobutyric acid (GABA) transporters [20, 21]. They observed that KD ad libitum was able to reverse the age-dependent decrease of vesicular GABA transporter expression in the hippocampus and in the prefrontal cortex. This result could be linked to the known effect of KD in epilepsy [3]. Indeed, KD enhances GABA vesicular filling which maintains an inhibitory feedback associated with the beneficial effect of KD. One can also hypothesize that this effect on GABA may have a potential anxiolytic effect. Interestingly, the authors described a similar reversal effect on the age-dependent decrease of vesicular glutamate transporter expression that was not restricted to the hippocampus.

In addition, Wang and Mitchell described that KS is associated, in old WT rats, with an increase in insulin receptor 1 levels and Akt phosphorylation and a decrease in ribosomal protein S6 kinase phosphorylation [27]. KS effect on this pathway is associated with increased ube3a expression that notably plays a role in synaptic stabilization. Conversely, Newman et al. identified a downregulation of the insulin pathway in old WT mice fed with KD ad libitum [23]. The conflicting results of the two studies can be due to the discrepancies concerning the duration of diet (8 vs 72 weeks) and/or the baseline age of the animals (21 vs 12 months). So far, data are not sufficient to conclude on the mechanisms responsible for the cognitive impact of KD/KS in ageing brain or whether there even is an impact. However, if there is an effect of KD/KS on cognition, one first hypothesis might be synaptic protection through neurotransmitter pathway and/or synaptic stabilizations.

#### Effects of KD/KS on weight

Two studies [20, 27] showed a significant weight loss while no body weight modifications were observed in the two others [23, 24]. Among the four, only Hernandez et al. observed a significant weight loss despite a calorie-controlled KD. This effect was specific to old WT rats, but an unexpected fat mass reduction was observed both in young and old WT rats [20].

### In Alzheimer’s disease animal models

In details, five studies using AD mouse models assessed cognition and/or motor function together with histological data [17–19, 22, 26] while one assessed both impacts on weight and histology [24].

### Cognitive and motor impact of KD/KS

Two studies assessed the cognitive impact of KD in AD mouse models [17, 22]. Mice only showed a cognitive gain in the latter study, in which triheptanoin was added to KD. According to the authors, the cognitive gain was likely to result from an enhanced mitochondrial function, due to triheptanoin supplementation [17]. In the other study, motor coordination was significantly improved in the KD group.

#### Effects of KD/KS on AD pathological lesions

Five studies specifically assessed the changes in Aβ and/or tau deposition in AD mouse models [17–19, 22, 26]. Kashiwaya et al. and Van der Auwera et al. described a decrease in Aβ deposition in the brain of KD-fed female APP [V7171] and male 3xTgAD mice, respectively. In addition, Kashiwaya et al. reported a significant reduction of abnormal pTau labelling in the hippocampal neurons of 3xTgAD mice under a calorie-controlled KD. On the other hand, Aso et al., Beckett et al. and Brownlow et al. failed to report any significant improvement in brain amyloid load after KD in APP/PS1 [17, 18], APP+PS1 and Tg4510 (tau) AD mouse models [19].

Several factors or combined factors could explain this discrepancy, but we will focus on the two mains: (i) type of diet and/or its duration and (ii) type of AD model and time course of lesion onset. Regarding diet, the duration of the intervention might have played a role in the observed discrepancy. Indeed, this effect on Aβ accumulation and pTau pathology in 3xTgAD mice was described when an animal received a KS for 32 weeks [22]. This result may also be due to a specific therapeutic effect of the synthetic ketone ester (R)-3-hydroxybutyrate-(R)-1,3-butanediol monoester, administered as a dietary supplement.

The choice of an AD mouse model is crucial in this setting. All AD mouse models used are based on the overexpression of Aβ, but they differed in terms of the type and/or the pathological time course of the disease. Thus, promoters regulating the mutated gene expression are model-dependent and so is Aβ production and accumulation. AD models with high Aβ production and accumulation like 3xTgAD (combining amyloidopathy and tauopathy) or APP [V7171] (early-onset familial AD) were the ones in which the effect of the ketogenic interventions was observed [22, 26]. KD, like calorie-restricted diet, might induce insulin-degrading enzymes directed against Aβ [42]. Accordingly, an effect on Aβ accumulation based on degradation is easier to observe in animals with higher Aβ accumulation. In addition, the time course of the lesions depends on model types. Therefore, if the aim of the intervention is to evaluate the effect on Aβ load, the intervention has to take into account this parameter. For example, in APP/PS1 mice, soluble Aβ became detectable from 3 months onward and amyloid plaques appeared at 9 months. Studying the effect of KD/KS before those milestones or with an early endpoint may lead to the absence of observed effect [17, 18]. It is also interesting to note that the type of promoter also affects the magnitude of Aβ load. For example, the prion protein promoter, present in APP+PS1 and APP/PS1, is responsible for a more widespread and less selective Aβ expression than others [43]. Localization of amyloid brain load may differ from one animal model to the other, and analyzing methods have to be adapted. Thus, analysis restricted to specific brain regions (e.g. Aβ immunochemical labelling in the prefrontal cortex or hippocampus [24]) seems to be more efficient than global analysis (e.g. whole-brain enzyme-linked immunosorbent assay against total Aβ and APP C-terminal [25]).

Regarding other abnormal features in models of AD, Aso et al. showed a reduction of neuroinflammation after intake of KD + triheptanoin in APP/PS1 mouse that was not observed in APP/PS1 mouse fed with KD only and thus might be specific to triheptanoin supplementation [17]. Pawlosky et al. observed that KS induced a reduction of the free radical insult against hippocampal proteins and modified both α- and β-secretase expression levels (higher and lower respectively) in hippocampal but not in cortical lysates [25]. KS is able to reduce the levels of hippocampal BACE 1, a key enzyme in the production of Aβ peptide, and this finding could explain the reduction of amyloid load.

Overall, KD and KS seemed to be able to reduce Aβ and pTau load as well as neuroinflammation, especially in aggressive mouse models treated for a long time. In some models, those neuropathological effects were associated with improvement of cognitive and/or motor functions. While the underlying mechanisms remain to be elucidated, these observations support the hypothesis that KD/KS might have definite therapeutic actions on AD abnormal biological pathways.

#### Effects of KD/KS on weight

Four studies, using various methodologies, observed a significant weight loss [18, 22, 25, 26], while no body weight modifications were observed in the last one [19]. Thus, while these studies used variable material and approaches, most of them found a reproducible weight loss induced by KD or KS. However, details are lacking to draw definite conclusions on the mechanisms of this weight loss and its consequences on the safety of this approach. There was no reported association of weight loss to cognitive change in these studies.

## Clinical studies

### General characteristics of the studies

Included humans studies (*N* = 11) are presented in Table 2. They involved either ageing subjects with or without mild cognitive impairment (MCI) (*N* = 6; mean age 66.1 to 75.4 years old; mean Mini-Mental Status Examination (MMSE) score at baseline 17.1 to 27.1) or individuals with AD dementia based on clinical diagnostic criteria such as the Diagnostic and Statistical Manual of Mental Disorders-IV or the National Institute of Neurological and Communicative Diseases and Stroke/Alzheimer’s Disease and Related Disorders Association (*N* = 5). In addition, these studies comprised inconsistent sex ratios (30 to 66% of male). Five of them indicated an average level of education between 12.5 and 15.3, and only one provided information about the ethnic groups of the study population (mostly Caucasians in 91%).

Thus, participants showed various degrees of cognitive performance among studies, from normal ageing people to moderate dementia. Two studies did not specify the degree of cognitive impairment of their participants [33, 36]. One can notice that the use of Aβ or tau biomarkers, highly recommended for increasing diagnostic accuracy, was not mentioned in these studies. APOE genotyping was performed in four studies only [28, 30, 37, 38].

Regarding nutritional interventions, two studies used a classic KD approach while 11 relied on KS using MCT. Of note, KS protocols differed much in composition and daily dose (20 to 56 g of MCT per day). Two studies had a crossover design [29, 38] while one did not include a control group [31]. The seven remaining studies were randomized controlled trials. The number of individuals included varied among studies from 6 to 152 (mean 31.3 ± 38.2). Likewise, the follow-up durations were rather short and heterogeneous, from 0 to 26 weeks (mean 11.2 ± 9) including three studies examining the immediate effect, the same day as the clinical and cognitive examination of KS [28, 30, 37, 38].

None of the studies included in this review provided details on patients’ medical conditions. Apart from cognitive impairment, comorbidities are likely to modulate the efficacy or the adhesion to KD or KS. For instance, in patients with type 2 diabetes, very stringent KD may not be sustainable over long-term, although there is no formal contraindication to it [44]. Besides inflammatory diseases, mood disorders, chronic pain and polypharmacy may change the nutritional status of the individuals, as well as the safety of a high-fat diet. Moreover, only Abe et al. presented its exclusion criteria: subjects with body mass index < 23 kg/m^2^ or major organ dysfunction [32]. Altogether, this impairs the external validity of the studies and pleads for new studies that would assess the interaction between comorbidities and KD/KS in patients.

### Outcomes

All studies but one assessed cognitive changes after KS or KD. One study focused on cognitive changes and the feasibility of long-term KS in cognitively impaired individuals [29]. However, the instruments used to assess cognitive outcomes were inconsistent among studies. Five studies used the Mini-Mental Status Examination [28, 31, 32, 34, 38], and four of them utilized the Alzheimer Disease Assessment Scale (ADAS-cog) [31, 35, 37, 38]. Two studies used a neuroimaging (PET) endpoint: regional cerebral blood flow [30] or [11C]-acetoacetate tracer [28].

#### Metabolic effects of KD/KS in cognitively impaired patients and older adults

Nine studies out of eleven monitored the plasma levels of KB to make sure that participants actually achieved ketosis. Thus, the authors were able to demonstrate that ketosis was quickly and efficiently reached in all the studies, regardless of the type of intervention. However, only five studies examined the changes in nutritional status or body composition in patients under KS/KD [28, 31, 32, 35, 36]. Krikorian et al. highlighted a mean weight loss of 4 kg per individual after 6 weeks, in MCI adults under KD as compared to patients under control diet [36]. This finding is consistent with the results of a meta-analysis showing that KD is commonly associated with weight loss, due to reduced total calorie intake [45].

Three other studies did not show any significant weight change with KS [28, 31, 35]. Interestingly, Abe et al. observed that participants improved their muscle mass and function [32]. Of note, those subjects received vitamin D and leucine in addition to the nutritional intervention, which may have contributed to this anabolic effect.

Finally, only four studies reported on the side effects of KS/KD [28, 31, 36, 37], including the study by Henderson et al. that described gastrointestinal symptoms (49%), which led to treatment discontinuation (23% in the intervention group).

#### Cognitive effects of KD/KS

Apart from the Torosyan et al. study (no clinical endpoint) [30], the majority of the studies (six out of ten) observed significant cognitive improvements in the intervention groups (KS or KD), regardless of participants’ cognitive status (from MCI to severe AD patients). On the other hand, the three remaining studies did not report any significant effect on cognition [28, 34, 35]. Cunnane et al. previously mentioned that given a choice between glucose and ketones, neurons would rather consume the latter [8]. Besides, even though brain-imaging studies revealed that brain utilization of glucose declines in early AD, KB utilization does not [46]. The results of the two PET imaging studies evaluated for this review were consistent with these findings [28, 30]. Interestingly, Torosyan et al. showed a long-term increase in brain metabolism after KS in non-APOE4 individuals [30]. This is in line with the results of three studies included in this review that highlighted better efficiency of KS in non-APOE4 subjects [30, 37, 38]. There is also an established relationship between APOE4 genotype and brain amyloid deposition. It is noteworthy to say that in the PREDIMED-NAVARRA randomized-controlled trial, non-APOE4 genotype was associated with greater improvement of the MMSE and Clock Drawing Test scores when following a Mediterranean diet rather than a Western-type diet [47]. Thus, the relationship between APOE4 status and cognitive effects of metabolic interventions deserves further investigations.

### Limitations of the studies

Despite the interesting findings of these 13 studies, many limitations must be acknowledged. First, studies appear highly heterogeneous, in particular regarding age, gender ratios or participants’ cognitive status, which all have a significant effect on the risk of subsequent cognitive decline. These limitations prevent from drawing robust conclusions about the cognitive benefits of the ketogenic interventions. Some results may also be questionable such as an unexpected cognitive decline measured with the ADAS-cog scale under placebo after only 45 days in MCI individuals whereas in the intervention group, participants maintained their cognitive level [37].

The short follow-up durations and the repeated cognitive assessments are likely to be responsible for a retest effect especially in cognitively intact or MCI individuals. Conversely, patients with mild-to-moderate dementia may be too severely impaired to observe benefits from an intervention. This observation was previously raised when discussing the failure of anti-amyloid therapies in AD [48]. Furthermore, all studies aimed at measuring short-term changes in cognition or brain metabolism. The absence of long-term follow-up did not provide any insight into the persistence of cognitive changes after discontinuation of the nutritional intervention. Adhesion to the KD or to KS intake must be carefully examined as far as long-term nutritional changes are expected. Besides, the monitoring of potential adverse effects is mandatory, especially in AD, since nutritional status is a key predictor of rapid cognitive decline as well as functional limitations [49]. Finally, the small number of studies published so far raises the issue of a potential publication bias as negative studies on the effects of KS or KD on cognition could not be published.

## Conclusion

Despite the growing interest for KD in AD over the last years, only few interventional studies in animals or humans clearly addressed the subject. In preclinical studies, this review pointed out some interesting results such as improvement of cognition and motor function in some AD mouse model or ageing animals. The mechanisms leading to a benefic effect on cognition could be due to the (i) modification in neurotransmitter transport pathway and/or synaptic maintenance in ageing WT animals and (ii) improvement of abnormal features (Aβ load or neuroinflammation) in AD mouse models. However, we must keep in mind that KD was initially created (and is still popular) for inducing weight loss in healthy or overweighed adults. In cognitively impaired subjects, older adults or animal studies, it was also often associated with a significant weight loss. This consequence could play an adverse effect on muscle performance (sarcopenia) or even on cognitive decline. In humans, notwithstanding the high heterogeneity of the studies and methodological issues discussed above, most of the published studies could suggest improved cognitive outcomes (memory, executive function or global cognition) with KS or KD, regardless of the severity of cognitive impairment. Both interventions seemed acceptable for included subjects and show efficacy to achieve ketosis. KD and KS intake both offer different features and potential benefits. They also present different disadvantages. KD might be difficult to start in older adults with modern eating habits and even more difficult to maintain. KS are useful to achieve ketosis but do not lead to the same metabolic shift, since the brain is still being fueled by glucose.

As a conclusion, the KD might be a promising way to fight against the cognitive symptoms of AD, especially from the prodromal stage of the disease (MCI). Regarding the body of evidence discussed above, the hypothesis that KD could postpone cognitive decline in AD should be explored. Unlike omega-3 supplements or *Ginkgo biloba*, the ketogenic interventions have not been evaluated yet in large sample randomized controlled trials with sufficient follow-up and structured cognitive and neuroimaging outcomes. Therefore, further studies are warranted, in particular, in adults with early AD, not only to assess the efficiency of the KD on cognitive decline, but also to examine the adverse effects (e.g. weight loss, malnutrition) as well as the adherence to the diet.

## Data Availability

Not applicable.

## Abbreviations

Aβ: β-Amyloid peptide
AD: Alzheimer’s disease
ADAS-cog: Alzheimer Disease Assessment
ADCS-CGIC: Alzheimer’s Disease Cooperative Study Scale Clinical Global Impression of Change
APOE4: Apolipoprotein E allele 4
APP: Amyloid precursor protein
BACE 1: Beta-secretase 1
cal ctrl: Calorie-controlled
CD: Control diet
DST: Digit Symbol Test
FDG: Flurodeoxyglucose
FU: Follow-up
GABA: Gamma-aminobutyric acid
GI: Gastro-intestinal
KB: Ketone bodies
KD: Ketogenic diet
KS: Ketone supplementation
MCI: Mild cognitive impairment
MCT: Medium-chain triglyceride
MeSH: Medical subject heading
MMSE: Mini-Mental Status Examination
MoCA: Montreal Cognitive Assessment
PET: Positron emission tomography
PS: Presenilin
pTau: Phosphorylated tau
RCT: Randomized controlled trial
TMT: Trail Making Test
vitD: Vitamin D
WT: Wild-type
